# Dopaminergic Control of Inflammation and Glycemia in Sepsis and Diabetes

**DOI:** 10.3389/fimmu.2018.00943

**Published:** 2018-05-04

**Authors:** Eleonora Feketeova, Zhifeng Li, Biju Joseph, Roshan Shah, Zoltan Spolarics, Luis Ulloa

**Affiliations:** Department of Surgery, Center for Immunity and Inflammation, Rutgers-New Jersey Medical School, Newark, NJ, United States

**Keywords:** diabetic sepsis, inflammation mediators, dopaminergic agonist, murine sepsis, phosphorylation

## Abstract

Most preclinical treatments for sepsis failed in clinical trials in part because the experimental models of sepsis were performed on healthy animals that do not mimic septic patients. Here, we report that experimental diabetes worsens glycemia, inflammation, and mortality in experimental sepsis. Diabetes increases hyperglycemia, systemic inflammation, and mortality in sepsis. Diabetes exacerbates serum tumor necrosis factor (TNF) levels in sepsis by increasing splenic TNF production. Both serum from diabetic mice and glucose increase cytokine production in splenocytes. Anti-inflammatory treatments cannot control hyperglycemia and are less effective in diabetic patients. By contrast, dopaminergic agonist type-1, fenoldopam, attenuates hyperglycemia, and systemic inflammation in diabetic septic mice by inhibiting splenic p65NF-kB phosphorylation. Fenoldopam inhibits TNF production in splenocytes even at high glucose concentrations and inhibits the canonical NF-kB pathway by inhibiting p65RelA and p50NF-kB1 phosphorylation without affecting the non-canonical NF-kB proteins. Treatment with fenoldopam *rescues* diabetic mice from established polymicrobial peritonitis even when the treatment is started after the onset of sepsis. These results suggest that dopaminergic agonists can control hyperglycemia, systemic inflammation and provide therapeutic advantages for treating diabetic patients with sepsis in a clinically relevant time frame.

## Introduction

The regulation of the immune system and inflammation is critical for survival both from a physiological and a clinical perspective. Probably one of the most characteristic examples is sepsis, a major clinical challenge in modern medicine killing around 250,000 patients every year and accounting for 9.3% of overall deaths in the United States (1–6). Sepsis was originally defined as a systemic infection and its diagnosis required the confirmation of bacterial infection. Thus, initial strategies focused on designing effective antibiotics to control the infection. New generations of antibiotics are more effective controlling infections, but sepsis still causes around 1/3 deaths in hospitalized patients with high mortality rates in the ICU ranking from 30 to 60% depending on the clinical study and the organ failure (3–6). In addition to the infection, septic is also characterized by detrimental systemic inflammatory responses that become more dangerous than the original infection and cause organ damage and lethal multiple organ failures (7–11). The inhibition of specific inflammatory cytokines such as a tumor necrosis factor (TNF), migration inhibitory factor (MIF), or high mobility group box (HMGB)-1 provided promising results in experimental sepsis (7, 10–13), but, they failed in the clinical trials for sepsis (14). One explanation is that sepsis is not produced by a single cytokine and thus, successful treatments for sepsis may require inhibiting multiple cytokines. Therefore, recent efforts focus on designing therapeutic strategies that control multiple inflammatory factors and rescue patients from established sepsis in a clinically relevant time frame.

Most preclinical strategies that provided promising results in experimental models of sepsis, failed in clinical trials (7, 10). Indeed, more than 100 randomized clinical trials tested whether inhibition of inflammatory factors improves survival in sepsis. With one short-lived exception, none of these clinical trials have resulted in new treatments (15). Xigris [activated protein C (APC), drotrecogin-alpha, DrotAA] was the only drug approved by the FDA for treating severe sepsis, as it improved survival by 6% in the 2001 PROWESS trial. However, Xigris increased the risk of severe hemorrhage in septic patients, and Eli Lily withdrew it from the market in 2011 due to the lack of beneficial effects in the PROWESS-SHOCK trial (16). Currently, there is no treatment for severe sepsis approved by the FDA and present therapies are mostly supportive. Current studies indicate that the pathology of sepsis is a complex process with both immune and metabolic alterations, and most septic patients have preexisting conditions with metabolic and immune alterations that contribute to multiple organ failure in sepsis (17–25). Thus, one potential reason for the failure of these clinical trials is that the preclinical studies focused on healthy animals that did not mimic the preexisting conditions of septic patients (26). The CDC reported that 7 in 10 septic patients had chronic diseases requiring frequent medical care or required hospital services 30 days before sepsis admission (27). Indeed, around 1/3 of septic patients are diabetic, and hyperglycemia increases 90-day mortality in septic patients (17–29). There is a huge population of prediabetic patients and as many as 50% of diabetic patients can be asymptomatic and remain undiagnosed (30). Diabetic patients also represent an additional challenge because insulin treatment is not effective during sepsis (31). Sepsis is characterized by detrimental inflammatory and hyperglycemic responses to infection (32), and this combination is associated with higher mortality rates over 40% (32–34). Despite the use of new generations of antibiotics and regardless of their higher susceptibility to infection, diabetic patients have a higher mortality rate in sepsis. Thus, recent efforts focus on identifying the mechanisms connecting metabolic and immune alterations and their clinical implications in infectious and inflammatory disorders. Here, we analyze how experimental diabetes affects sepsis and the efficacy of anti-inflammatory treatments for sepsis.

## Materials and Methods

### Chemicals and Reagents

LPS (*Escherichia coli* 0111:B4), streptozotocin, glucose, dopamine hydrochloride, and fenoldopam were purchased from Sigma-Aldrich^®^ (Saint Louis, MO, USA). The glucose measuring strips were purchased from PharmaTech Solutions, Inc. (Westlake Village, CA, USA). Pentobarbital sodium was purchased from Diamondback (Scottsdale, AZ, USA); ketamine from Henry Schein animal health (Dublin, OH, USA); xylazine from Akorn animal health (Lake Forest, IL, USA), and enrofloxacin from Bayer Healthcare (Shawnee Mission, KS, USA). Streptozotocin was injected (STZ; i.p., 50 mg/kg) at 10 and 5 days before the experiment as previously reported (35, 36). Treatment with fenoldopam (Fen; 10 mg/kg/dose; i.p.) was administered at 6 and 1 h before LPS or CLP in most experiments. Treatment with fenoldopam was started 15 h after CLP and given every 12 h for 3 days in the survival experiments.

### Animal Experiments

All experimental procedures adhered to *The Guide for the Care and Use of Laboratory Animals* by the National Academy of Sciences and published by the National Institutes of Health (Copyright© 1996 by the National Academy of Sciences), and were approved by the Institutional Animal Care & Use Committee of the Rutgers New Jersey Medical School. 6–8-week-old (≈25 ± 5 g) BALB/c male mice obtained from Charles River Laboratories (Wilmington, MA, USA) were maintained in a controlled environment, room temperature 70–75 F, air humidity 40–70%, 12-h light/dark cycle, with free access to food and water (*ad libitum*) until experimentation. Animals were randomly distributed for the experimental treatments, and the investigators were blinded to the treatments.

### Experimental Sepsis

Endotoxemia and cecal ligation and puncture (CLP) were performed as we previously described in Nat Med (37) with the modifications described in Nat Med (38). *Endotoxemia*: Endotoxin (*E. coli* LPS 0111:B4; Sigma Chemical, Saint Louis, MO, USA) was dissolved in sterile, pyrogen-free PBS (Gibco^®^: Life Technologies, Grand Island, NY, USA), and sonicated for 20 min immediately before use. Animals received a LD_50_ dose of LPS (10 mg/kg, i.p.). CLP: animals were anesthetized with pentobarbital sodium (50 mg/kg, i.p.; Diamondback, Scottsdale, AZ, USA). Animals underwent to a standard CLP procedure with 25–50% average mortality as we described in Nat Med (37, 38). Briefly, an abdominal incision, of approximately 1.0 cm, was performed to expose and ligate the cecum at 5.0 mm from the cecal tip away from the ileocecal valve. The ligated cecal stump was punctured only once with a 22-gauge needle, and the stool was extruded (approx. 1.0 mm) to ascertain patency of puncture. The abdominal wound was closed in two layers, peritoneum and fascia separately, to prevent leakage of fluid. All animals received antibiotic (Enrofloxacin 2.5 mg/kg, s.c.; Baytril^®^, Bayer Health Care™, Swanee Mission, KA, USA) dissolved in 0.9% normal saline immediately after surgery and every 12 h for 3 days, 0.5 mL/dose.

### Splenectomy

Animals were anesthetized with rodent cocktail 100-mg/kg ketamine; 20-mg/kg xylazine; intraperitoneal. Anesthesia was confirmed by the absence of withdrawal reflex to toe pinch. Splenectomy was performed 3 days before the experimental procedure as we described in J Exp Med (39). Right after surgery, all animals received antibiotic (Enrofloxacin 2.5 mg/kg, s.c) dissolved in 0.9% normal saline immediately after surgery and every 12 h for 3 days. Anesthetized animals were subjected to an abdominal incision on the epigastrium and mesogastrium. The spleen was exposed by gentle retraction of the stomach to the side. The three main branches of the spleen artery were stabilized with nylon thread, ligated and cut. The spleen was removed and the wound was closed with sutures; catgut for the abdominal wall, and nylon thread for the skin.

### Cell Cultures

Primary culture of splenocytes and peritoneal macrophages were performed as we previously described (39). Murine RAW264.7 cells (ATCC, Manassas, VA, USA) were cultured as we previously described (37). Cells were transferred onto a 24-well polystyrene culture plates at 3 × 10^5^ cells/well and incubated overnight. Cells were washed with PBS and incubated overnight with 5% serum-free DMEM medium. Cells were incubated with DMEM, no glucose (ThermoFisher, SKU# 11966-025) supplemented with the indicated concentrations of glucose. Alternatively, cells were incubated directly on serum without dilution from normal or diabetic mice for 3 h prior LPS challenge. Cells were lysed in lysis buffer with protease inhibitor (CelLyticMT and Protease Inhibitor Cocktail P8340; 1:100 v/v, both from Sigma-Aldrich, Saint Louis, MO, USA) and centrifuged at 12,000× *g* for 20 min at 4°C for NF-kB analyses. The conditioned supernatant was used for TNF analyses.

### Blood, Organ, and Cell Analyses

Serum samples were obtained by clotting the blood for 2 h at room temperature, and centrifuged at 2,000× *g* for 15 min at 4°C. Organs were collected and immediately homogenized in 4°C PBS. Samples were normalized to protein concentration and TNF was analyzed by ELISA (Affymettrix Inc, San Diego, CA, USA). Glucose was analyzed from the mouse tail tip blood using the Genstrip (PharmaTech Solutions Inc., Westlake Village, CA, USA) and the Onetouch UltraMini glucometer (LifeScan Inc., Milpitas, CA, USA). TNF in the culture cells was analyzed at 3 h post-LPS. TNF levels in the serum and organs were analyzed at 90 min after the LPS treatment. Cell samples for NF-kB analyses were normalized to protein concentration and their activation and binding to DNA was analyzed using the TransAM DNA-Binding ELISA (Active Motif; Cambridge, MA, USA). Phosphorylation of p65NF-kB protein at serine 536 was analyzed by ELISA using the specific (Total/Phospho) Multispecies InstantOne™ ELISA Kit (Cat# 85-86083-11; ThermoFisher, Waltham, MA, USA).

### Statistical Analyses

All tests were performed using the GraphPad Prism Software^®^ (GraphPad Software, La Jolla, CA, USA). The sample size was determined using standard deviation values and power analyses of our previous studies on the vagal stimulation (39, 40). All data in the figures are expressed as the mean ± SEM. The student’s *t*-test (Mann–Whitney *U* test) was used to compare mean values between two experimental groups. Analyses of three or more groups were performed using the one-way ANOVA with multiple pair-wise comparisons. The time courses and pair-wise comparisons were analyzed with the two-way ANOVA for repeated measures. Pair comparisons in ANOVA non-parametric tests were *post hoc* adjusted with Tukey test (in equal sample sizes) or Bonferroni’s for multiple hypothesis testing. Normality and homogeneity of variance were confirmed using the Kolmogorov–Smirnov analysis. Statistical analyses of survival were determined using the log-rank (Mantel–Cox) test. *n* = sample size per group. *p* < 0.05 are considered statistically significant and represented as follows: ^#^ Student’s *t*-test, ^+^ one-way ANOVA, *two-way ANOVA, and ^§^survival log-rank test.

## Results

### Diabetes Worsening Inflammation and Survival in Sepsis

**Figure 1:**
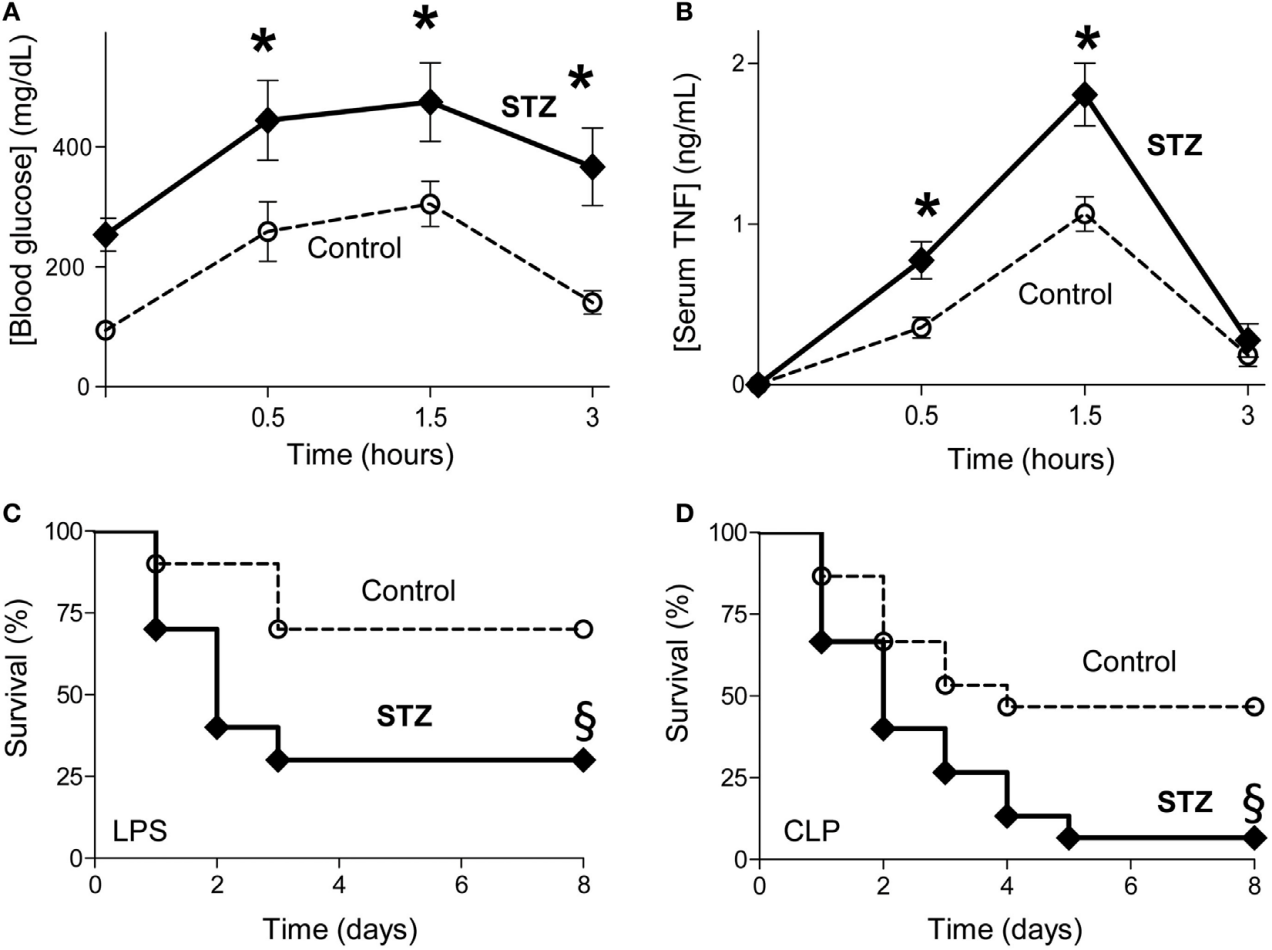
Diabetes worsening systemic inflammation and survival in sepsis. **(A,B)** Control (non-diabetic treated with vehicle solution) or diabetic [STZ; 40 mg/kg] mice were challenged with LPS (10 mg/kg; i.p., *n* = 4; *p < 0.05 vs. control, two-way ANOVA). **(A)** Blood glucose or **(B)** serum tumor necrosis factor (TNF) levels were analyzed at the indicated time points post LPS. **(C,D)** Kaplan–Meier survival analyses of control or diabetic (STZ) mice challenged with **(C)** endotoxemia (LPS, 10 mg/kg; i.p.; *n* = 10) or **(D)** polymicrobial peritonitis induced by cecal ligation and puncture (CLP; *n* = 15). ^§^*p* < 0.05 vs. control, survival log-rank test.

Given the high incidence of diabetes in septic patients, we first analyzed the effects of experimental diabetes on glycemia, inflammation and survival in sepsis. Diabetes was induced with streptozotocin, the standard and most common method for experimental diabetes (35, 36, 41). Treatment with streptozotocin increases blood glucose levels in mice by itself before the septic challenge (Figure 1A). By contrast, diabetes did not induce serum TNF levels before the septic challenge (Figure 1B). Furthermore, diabetes increases both hyperglycemia and serum TNF levels during endotoxemia (Figures 1A,B). Time course analyses show that diabetic and control non-diabetic animals have similar kinetics with glycemia and serum TNF levels peaks around 1.5 h and return to baseline after 3–4 h post-LPS. Next, we analyzed whether diabetes affects survival in different experimental models of sepsis including endotoxemia and polymicrobial peritonitis. Diabetes worsens survival in endotoxemic mice (Figure 1C). Diabetes also worsens the survival of mice with polymicrobial peritonitis induced by CLP, the standard experimental model to induce polymicrobial peritonitis (Figure 1D) (4, 42, 43). Unlike endotoxemia induced by LPS, CLP causes both polymicrobial infection (induced by the cecal puncture) and inflammation (induced by both the infection and the necrotic tissue of the cecal ligation) (4, 44). Diabetes decreased acute survival but the late deaths did not occur among control or STZ treated mice. These results indicate that diabetes worsens hyperglycemia, systemic inflammation and survival in experimental sepsis.

### Diabetes Enhancing Splenic TNF Production

**Figure 2:**
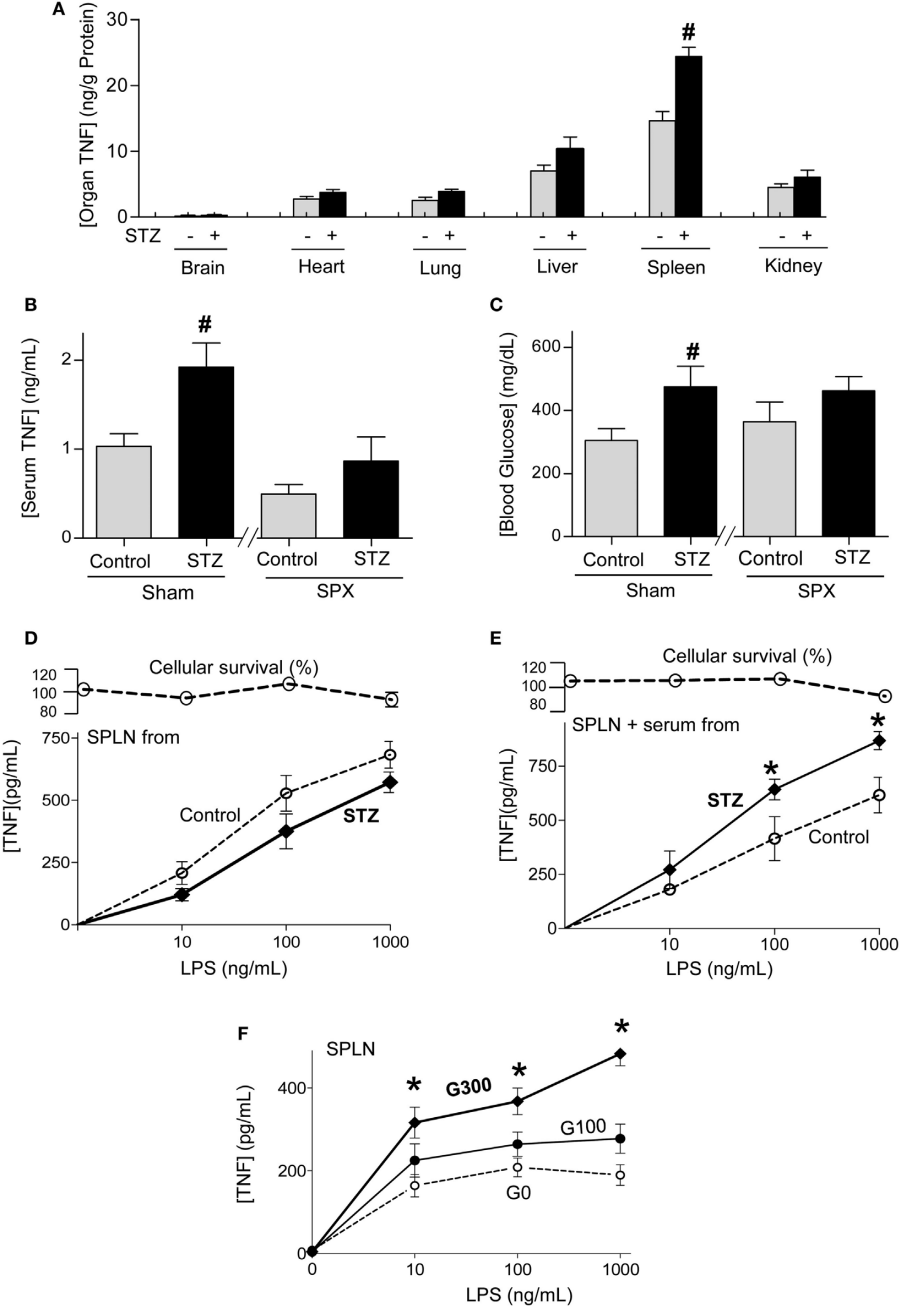
Diabetes increasing splenic tumor necrosis factor (TNF) production in sepsis. **(A)** Control or diabetic (STZ) mice were challenged with LPS (10 mg/kg; i.p., *n* = 4) and organ TNF concentrations were analyzed at 1.5 h post-LPS. **(B,C)** Mice underwent sham or surgical splenectomy (SPX) 3 days before LPS. **(B)** Serum TNF or **(C)** glucose levels were analyzed at 1.5 h post-LPS. ^#^*p* < 0.05 vs. control (*n* = 4/group, one-way ANOVA). **(D)** Primary culture of splenocytes from control or diabetic mice were challenged with LPS. **(E)** Primary cultures of splenocytes from untreated mice were incubated with serum from control or diabetic mice for 3 h, and then challenged with LPS. **(F)** Primary culture of splenocytes from untreated mice were incubated with different concentrations of glucose and challenged with LPS. **(D–F)** TNF levels in the conditioned culture media were analyzed at 3 h post-LPS. *p < 0.05 vs. control (*n* = 4, two-way ANOVA).

Next, we studied how experimental diabetes increases systemic inflammation in sepsis by analyzing TNF production in the organs. Bacterial endotoxin induces TNF production in all the organs but the highest TNF concentrations were found in the spleen (Figure 2A). Likewise, diabetic endotoxic mice have similar TNF levels in all the other organs but around twofold higher splenic TNF levels than non-diabetic endotoxic mice. These results suggest that diabetes increases serum TNF levels by increasing TNF production in the spleen. Thus, we analyzed whether the spleen is essential for the higher serum TNF levels in diabetic mice by performing surgical splenectomy 3 days prior endotoxemia. Diabetic mice have around twofold higher serum TNF levels than non-diabetic sham mice, but both diabetic and non-diabetic endotoxemic mice have similar serum TNF levels after splenectomy (Figure 2B). These results show that the higher serum TNF levels in diabetic mice are due to the higher TNF production in the spleen. Next, we analyzed whether splenectomy affects hyperglycemia. Splenectomy rendered diabetic and non-diabetic mice with similar hyperglycemia in endotoxemia (Figure 2C). Given that the higher serum TNF levels of diabetic mice are mainly due to higher splenic TNF production, we analyzed whether splenocytes from diabetic mice produce more TNF than those from non-diabetic mice. Primary culture of splenocytes from diabetic or non-diabetic mice produce similar TNF levels when challenged with LPS (Figure 2D). Thus, we analyzed whether the serum from diabetic mice enhances TNF production in splenocytes from non-diabetic mice. We isolated primary culture of splenocytes from normal mice and incubated them with serum from diabetic or non-diabetic mice before the endotoxic challenge. Serum from diabetic mice increases TNF production in primary culture of splenocytes from normal non-diabetic mice (Figure 2E). Thus, we reasoned that higher levels of glucose in the serum of diabetic mice enhance TNF production in splenocytes. We incubated primary culture of splenocytes from normal mice with different concentrations of glucose before the endotoxic challenge. Higher glucose concentrations enhance TNF production in normal splenocytes (Figure 2F). These results indicate that higher glucose concentrations in the serum of diabetic mice enhance the cytokine production in splenocytes and thereby worsens the prognosis of sepsis.

### Dopaminergic Type-1 Agonist Inhibiting TNF Production at High Glucose Concentrations

**Figure 3:**
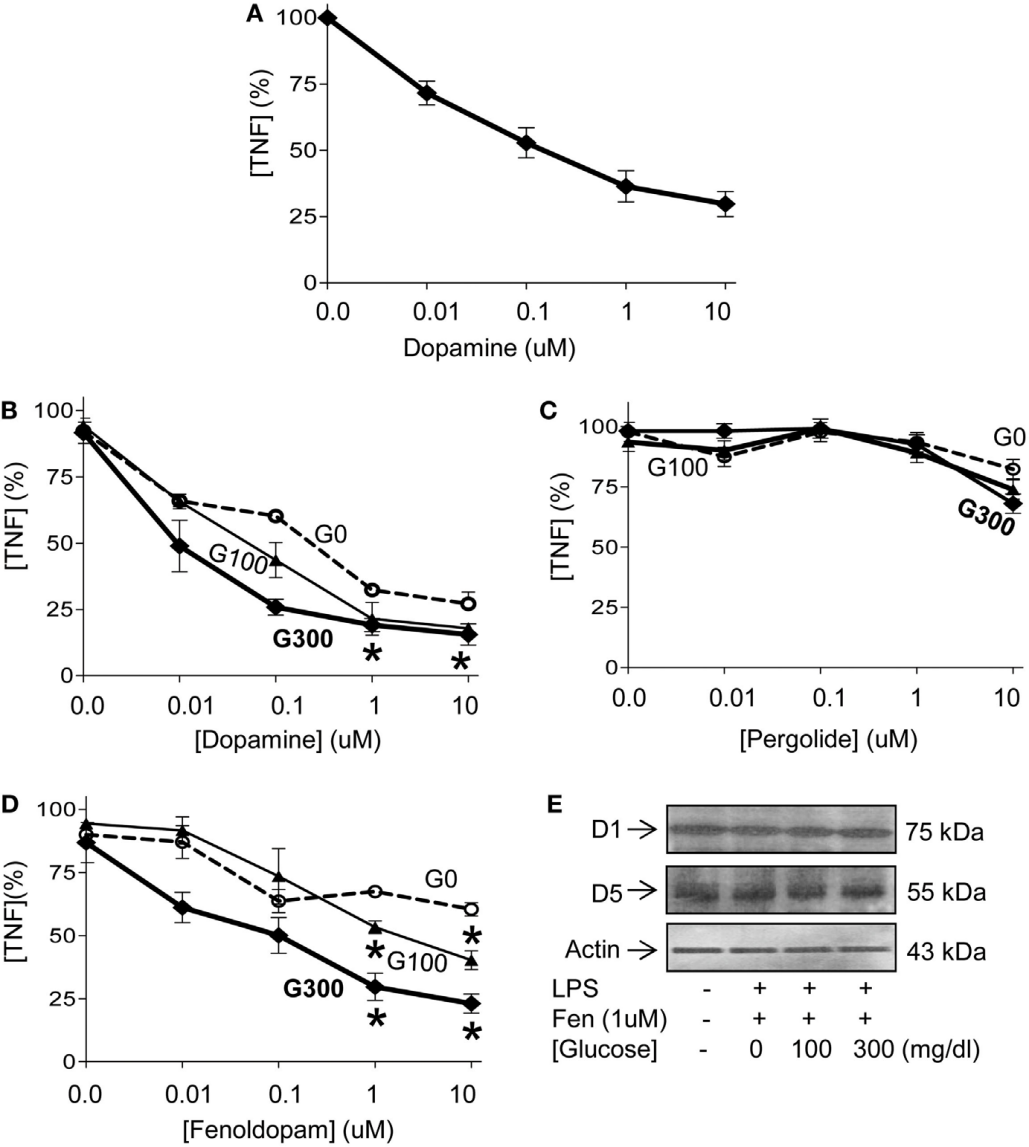
Glucose enhancing the dopaminergic anti-inflammatory pathway. **(A)** Primary culture of splenocytes were incubated with a concentration range of dopamine for 1 h prior LPS (100 ng/mL). Tumor necrosis factor (TNF) levels were analyzed in the conditioned culture media at 3 h post-LPS. **(B)** Primary culture of splenocytes were incubated with glucose [(G0) 0, (G100) 100 or (G300) 300 mg/dL] for 12 h and a concentration range of **(B)** dopamine, **(C)** pergolide, or **(D)** fenoldopam for 1 h prior LPS (100 ng/mL). TNF levels in the conditioned culture media were analyzed at 3 h post-LPS. *p < 0.05 of G100 or G300 vs. G0 (*n* = 4/group, two-way ANOVA). **(E)** Primary culture of splenocytes were incubated with glucose (0, 100, or 300 mg/dL) for 12 h. Then, treated with fenoldopam (1 µM) for 1 h prior LPS (100 ng/mL). Cells were harvested at 3 h post-LPS and dopaminergic receptors D1 and D5 were analyzed by Western blots. β-Actin was used to normalize protein loading.

Given that hyperglycemia increases inflammation and worsens the prognosis of septic patients (17–24), we reasoned that it may interfere with the efficacy of anti-inflammatory strategies for sepsis. We previously reported that electrical stimulation of the vagus nerve attenuates serum TNF levels in endotoxemia by activating the adrenal medulla to produce dopamine (38). Thus, we analyzed the potential of dopamine to inhibit TNF production in splenocytes. Dopamine inhibits TNF production in splenocytes in a concentration-dependent manner with a half maximal effective concentration (EC_50_) of 0.12 ± 0.3 µM (Figure 3A). Then, we analyzed whether extracellular glucose levels interfere with the potential of dopamine to inhibit TNF production in splenocytes. Low concentration of dopamine (0.1 µM) inhibits TNF production in splenocytes by around 55% and 70% at 100 and 300 mg/dL of glucose, respectively (Figure 3B). High concentration of dopamine (1 µM) inhibits TNF production in splenocytes by around 70% regardless of the concentration of glucose. Given that dopamine has multiple side effects as a pleiotropic factor signaling through multiple receptors, we used specific agonists to identify the receptors modulating cytokine production in splenocytes. According to IUPHAR nomenclature, dopamine signals through D1- and D2-like dopamine receptors. D1-like receptors include D1 and D5 dopaminergic receptors (42, 43, 45). D2-like receptors include D2, D3, and D4 dopaminergic receptors (46). Fenoldopam is a well-characterized D1-like agonist, whereas pergolide is the canonical D2-like agonist (42, 43, 45). D2-like agonist, pergolide fails to inhibit TNF production in splenocytes (Figure 3C). By contrast, D1-like agonist, fenoldopam inhibits LPS-induced TNF production in splenocytes even at high glucose concentrations (Figure 3D). Low concentration of fenoldopam (0.1 µM) inhibits TNF production by 35 and 50% at 100 and 300 mg/dL of glucose, respectively. High concentration of fenoldopam (1 µM) inhibits TNF production in splenocytes by 60 and 75% at 100 and 300 mg/dL of glucose, respectively. These differences were not due to changes in the expression of the receptors, and glucose did not affect the expression of dopaminergic receptor-1 or -5 (D1R, D5R) (Figure 3E). These results indicate that dopaminergic type-1 agonists such as fenoldopam can inhibit TNF production in splenocytes both at normal and high concentrations of glucose.

### Dopaminergic Control of the NF-kB Pathway in Sepsis

**Figure 4:**
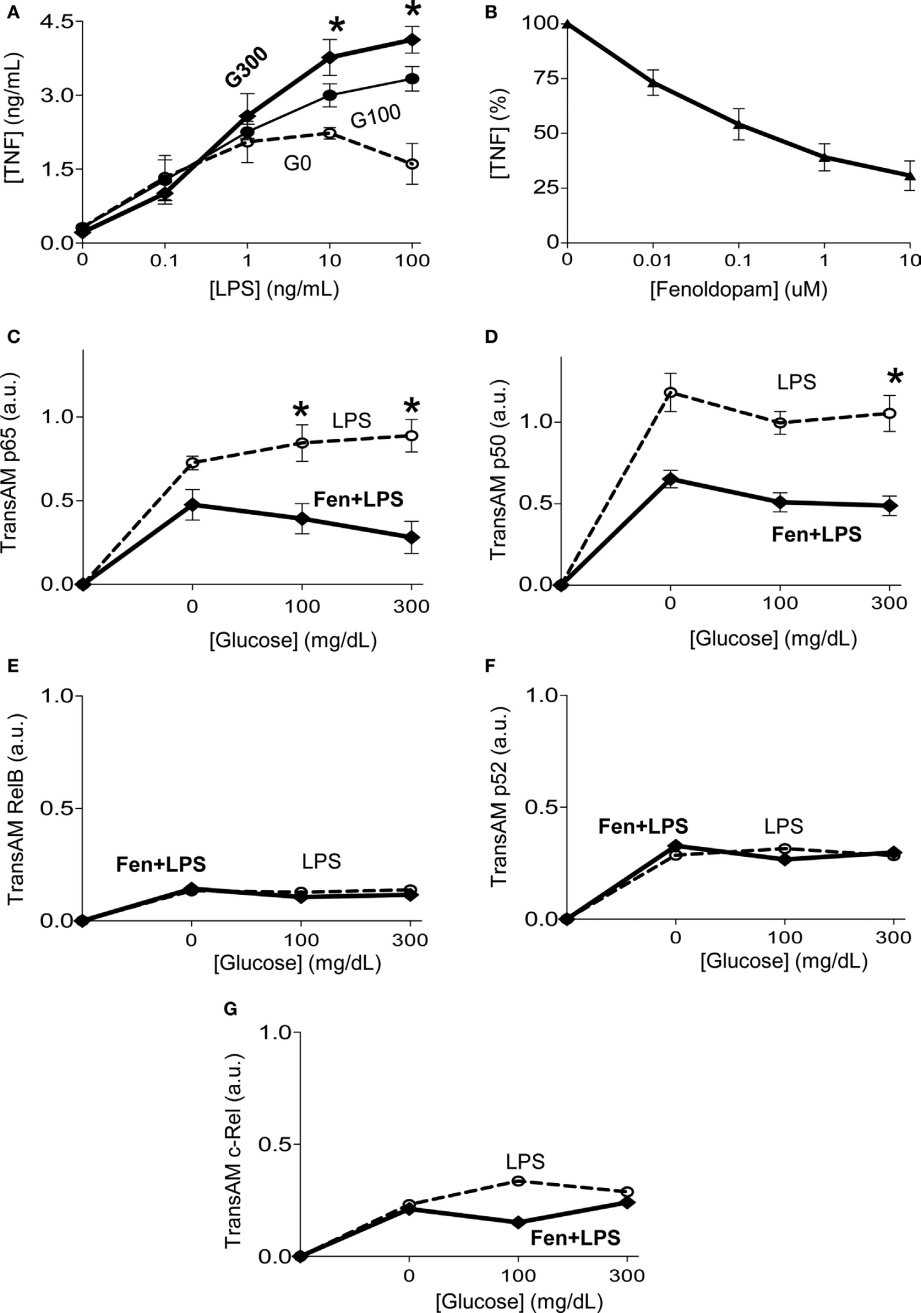
Glucose increasing dopaminergic control of the canonical NF-kB pathway. **(A)** RAW264.7 macrophage cells were incubated with glucose (0, 100, or 300 mg/dL) for 12 h, prior LPS (100 ng/mL). **(B)** RAW264.7 macrophage cells were incubated with fenoldopam for 1 h prior LPS, and tumor necrosis factor (TNF) levels were analyzed in the conditioned culture media at 3 h post-LPS. **(C–G)** RAW264.7 macrophage cells were incubated with glucose (0, 100, or 300 mg/dL) for 12 h. Then, treated with fenoldopam (1 µM) for 1 h before the LPS challenge (100 ng/mL). Cells were collected 30 min post-LPS, samples were normalized to protein concentration and DNA-binding of NF-kB **(C)** p65RelA, **(D)** p50, **(E)** RelB, **(F)** p52, or **(G)** c-Rel was analyzed by TransAM ELISA. *p < 0.05 vs. control (*n* = 3, one-way ANOVA).

Next, we analyzed how fenoldopam inhibits TNF production in macrophages. Given the heterogeneity of cell types in primary culture of splenocytes, we focused on homogeneous cultures of RAW264.7 macrophage cells similar as we previously described (37). Bacterial endotoxin induces TNF production in a concentration-dependent manner, and high concentrations of glucose enhance TNF production in RAW264.7 cells similar to that described in splenocytes (Figure 4A). Fenoldopam also inhibits TNF production in RAW264.7 cells in a concentration-dependent manner similar to that described in splenocytes (Figure 4B). Given that NF-kB proteins are key transcriptional factors regulating inflammatory cytokines in macrophages, we analyzed whether glucose affects the potential of fenoldopam to regulate their binding to DNA (47). Fenoldopam (1 µM) inhibits LPS-induced p65RelA activation and binding to DNA with higher efficacy at high glucose concentrations (Figure 4C). Likewise, fenoldopam (1 µM) also inhibits p50NF-kB1 activation and binding to DNA at high glucose concentrations (Figure 4D). We also analyzed the specificity of this inhibition and their potential to regulate the non-canonical NF-kB proteins. Neither extracellular glucose levels nor fenoldopam affects the DNA-binding of the non-canonical NF-kB proteins RelB, p52NF-kB2 and c-Rel (Figures 4E–G). These results indicate that D1-like dopamine receptor agonists can inhibit the canonical NF-kB pathway by inhibiting both p65RelA and p50NF-kB1 activation and DNA-binding even at high glucose concentrations.

### Dopaminergic Control of Sepsis With Diabetes

**Figure 5:**
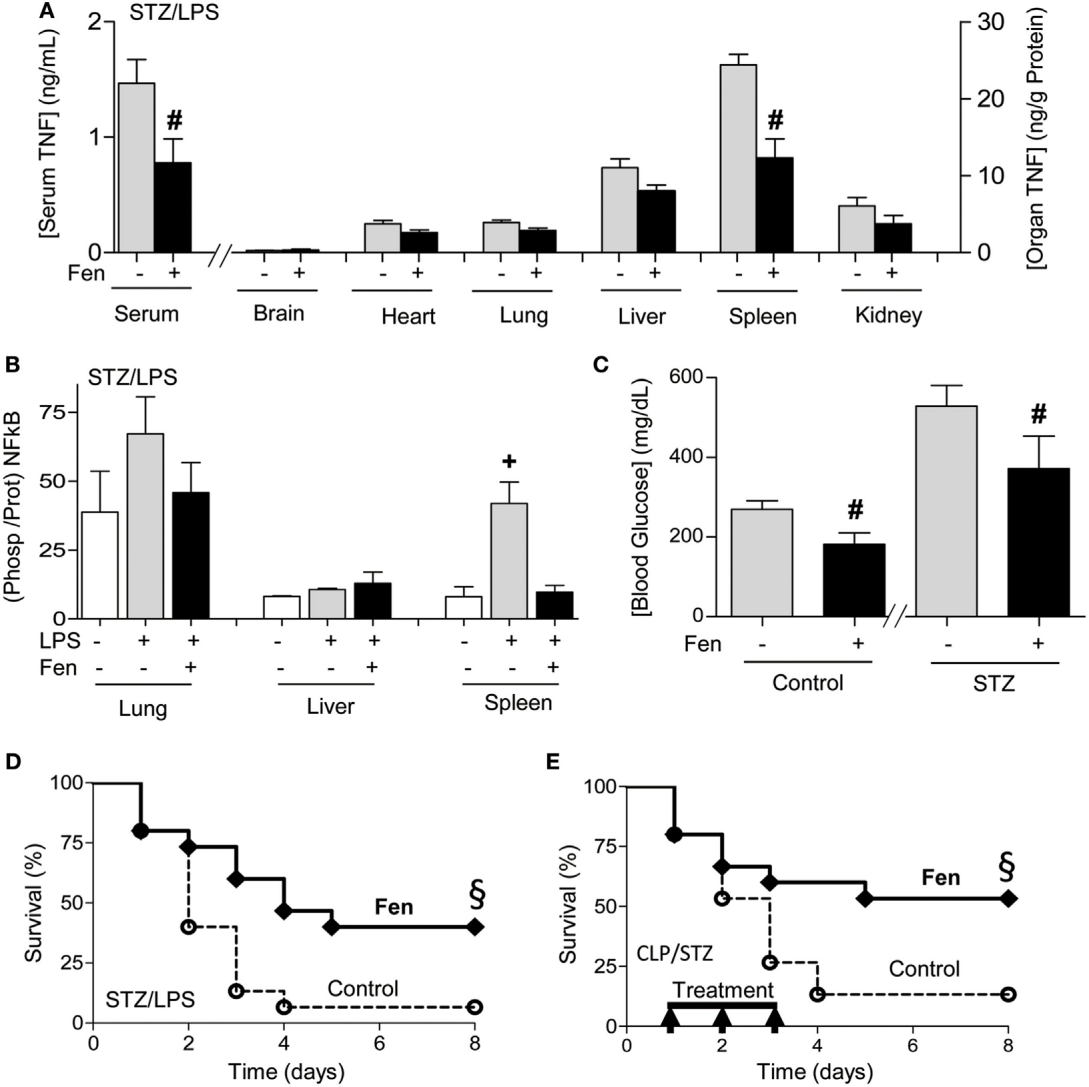
Dopaminergic agonist improving systemic inflammation and survival in sepsis with diabetes. **(A,B)** Diabetic mice were treated with vehicle or fenoldopam at 6 and 2 h prior LPS (10 mg/kg; i.p., *n* = 4) and **(A)** serum and organ tumor necrosis factor (TNF) concentrations, **(B)** p65RelA phosphorylation, and **(C)** blood glucose levels were analyzed at 1.5 h post-LPS. **(D,E)** Kaplan–Meier survival analyses of diabetic mice treated with vehicle (control) or fenoldopam (Fen, 10 mg/kg/dose; i.p., *n* = 15). **(D)** Mice were treated with fenoldopam 6 and 2 h prior LPS (LPS, 10 mg/kg; i.p.; *n* = 15). **(E)** Mice underwent cecal ligation and puncture and were treated with vehicle (control) or fenoldopam (Fen; 10 mg/kg/dose; i.p.). Arrows represent the treatments with fenoldopam (10 mg/kg/dose; i.p.) started 15 h after the CLP, and given every 12 h for 3 days. ^§^*p* < 0.05 vs. control (*n* = 13, survival log-rank test).

We next reasoned that dopaminergic agonists may have clinical implications for treating sepsis. Previous studies on sepsis are performed in experimental models of sepsis with “healthy” mice that do not mimic the preexisting conditions of septic patients (48). Given that around 1/3 of septic patients are diabetic and hyperglycemia increases 90-day mortality in septic patients (17–24), we analyzed whether fenoldopam attenuates systemic inflammation in experimental sepsis with diabetes. Treatment with fenoldopam attenuates serum TNF levels in endotoxemic mice with diabetes (Figure 5A). Then, we analyzed different organs to find that the most significant effects of fenoldopam were in the spleen by inhibiting TNF production by around 50% (Figure 5A). Given that NF-kB proteins are regulated by phosphorylation, we also analyzed p65NF-kB phosphorylation in the organs of septic mice with diabetes (Figure 5B). Again, the most significant effects were found in the spleen where endotoxin increases p65NF-kB phosphorylation at serine 536 by over fourfold, and fenoldopam inhibits this phosphorylation by over threefold in the spleen without affecting the lung or liver. Fenoldopam also attenuates hyperglycemia in both diabetic and non-diabetic mice (Figure 5C). Thus, we analyzed whether fenoldopam can improve survival in septic mice with diabetes. Treatment with fenoldopam at 6 and 2 h prior the LPS challenge improves survival in endotoxemic mice with diabetes (Figure 5D). Next, we analyzed whether fenoldopam can improve survival in diabetic mice with polymicrobial peritonitis induced by CLP, the standard experimental model to induce polymicrobial infection peritonitis (4, 44). Treatment with fenoldopam, started 15 h after the CLP, improves survival of diabetic mice with established polymicrobial peritonitis (Figure 5E). These results show that dopaminergic agonists can control systemic inflammation and improves the survival of diabetic mice in polymicrobial peritonitis.

## Discussion

Sepsis is a major clinical and scientific challenge in modern medicine with over 100 unsuccessful clinical trials (15). Many preclinical strategies improved survival in experimental animal models of sepsis but failed in clinical trials (7, 10). One explanation is that most experimental models of sepsis are performed on healthy animals that do not mimic the preexisting conditions of septic patients. Indeed, over 72% of the septic patients had chronic diseases requiring frequent medical care or required hospital services within 30 days before sepsis admission (27). This combination is associated with the highest mortality rates over 40% (32). Diabetes is a leading comorbidity in sepsis, around 1/3 of septic patient are diabetic, and hyperglycemia increases 90-day mortality in septic patients (17–30). Thus, experimental models of sepsis using “healthy” animals with “normal” blood glucose levels and physiological functions may not mimic the actual responses observed in septic patients. In our study, experimental diabetes was induced with streptozotocin, the standard method for experimental diabetes described in the literature (35, 36, 41). Streptozotocin induces both type-1 and type-2 insulin-resistant diabetes, causes DNA alkylation and activates poly ADP-ribosylation, leading to cellular NAD + and ATP depletion and the formation of superoxide radicals (49). Streptozotocin is a more specific, stable and reliable experimental model of diabetes that is neither diet dependent nor causes renal toxicity like alloxan (49). Our results show that diabetes increases serum glucose levels and induces hyperglycemia, but not detectable serum TNF levels by itself before the septic challenge. The effects of diabetes in sepsis are controversial. Some studies indicate that diabetic patients have functional immune deficiency and, they are less efficient in bacterial clearance (50–52). Likewise, experimental studies indicated that alloxan-diabetic mice are highly susceptible to polymicrobial sepsis due to downregulation of CXCR2 in neutrophils, preventing their migration to the focus of infection (53). However, despite the use of new generations of antibiotics, diabetic patients have a higher mortality rate in sepsis suggesting pathogenic effects regardless of their susceptibility to infection. Regardless of the infection, our results indicate that diabetes exacerbates both hyperglycemic and TNF responses to bacterial endotoxin. These results concur with previous studies indicating that diabetes exacerbates systemic inflammation and induces a persistent systemic inflammation in experimental sepsis (54–57). All these studies show that both type-1 and type-2 diabetic animals have exacerbated hyperglycemia, and production of both pro- (TNF, IL1, IL6, MCP1) and anti-inflammatory (IL10) cytokines in experimental sepsis. Our results also indicate that these exacerbated glycemic and inflammatory responses of diabetic mice worsen their survival in sepsis. Similar studies reported that alloxan-induced diabetes also worsens mice survival in polymicrobial peritonitis by preventing neutrophil migration to the focus of the infection (53). Regardless of the susceptibility to infection, our results indicate that diabetes increases mortality in endotoxemia but also in polymicrobial peritonitis induced by CLP even when the mice were treated with antibiotics to mimic clinical standards. Likewise, clinical studies show that diabetes and hyperglycemia increase morbidity and mortality in sepsis even using antibiotics (17–29). These results are not only relevant to sepsis, but an important clinical consideration as diabetic patients have exacerbated glycemia and inflammatory responses in critical conditions such as hemorrhage, ischemia and trauma.

Our results show that diabetes exacerbates the inflammatory responses to bacterial endotoxin by increasing TNF production in the spleen. Diabetic mice have around twofold higher splenic TNF levels than non-diabetic mice. However, both diabetic and non-diabetic mice have similar serum TNF levels after splenectomy. Of note, splenectomy increases hyperglycemia in non-diabetic but not in diabetic mice, rendering diabetic and non-diabetic mice with similar hyperglycemia in endotoxemia. Given that diabetes worsens systemic inflammation due to higher TNF production in the spleen, we analyzed the effects of diabetes at the cellular level in primary culture of splenocytes. Primary culture of splenocytes from either diabetic or non-diabetic mice produce similar TNF levels when challenged with bacterial endotoxin. These results suggest that acute diabetes does not produce a defect in the response of splenocytes to endotoxin. It remains to be determined if chronic diabetes, which is more clinically relevant, affects this response. However, serum from diabetic mice increases TNF production in primary culture of normal splenocytes. Our results indicate that glucose increases TNF production in splenocytes showing that hyperglycemia directly affects the inflammatory responses to bacterial endotoxin and contributes to systemic inflammation in sepsis.

Previous studies have focused on whether preexisting conditions such as diabetes increase susceptibility to infections and sepsis. Here, we analyzed whether diabetes affects the effectivity of potential treatments for sepsis. We previously reported that electrical vagal stimulation attenuates serum TNF levels in endotoxemia by activating the adrenal medulla to produce dopamine (38). The present study shows that dopamine (0.1 µM) inhibits TNF production in splenocytes even when cultured at high glucose concentration. Thus, dopamine may provide therapeutic advantages for treating diabetic patients with sepsis or other cri-tical conditions. Both dopamine and norepinephrine are commonly used in critically ill patients to restore tissue perfusion (58, 59). Although dopamine is more effective in improving renal hemodynamics (60), it has significant side effects, increases the risk of tachyarrhythmia (58, 59), and worsens survival in septic animals (61). We reasoned that these effects can be mediated by different dopaminergic receptors, and thus specific dopaminergic agonists may avoid the unspecific side effects. Given that dopamine signals through D1- and D2-like dopamine receptors (62–65). D1-like receptors include D1 and D5 dopaminergic receptors. D2-like receptors include D2, D3, and D4 dopaminergic receptors (46). D2-like agonist, pergolide did not affect TNF production. By contrast, D1-like agonist, fenoldopam inhibits TNF production in splenocytes similar to that reported by dopamine. Low concentration of fenoldopam (0.1 µM) inhibits TNF production in splenocytes cultured at high glucose concentration. At the cellular level, fenoldopam inhibits the activation of the canonical NF-kB pathway by preventing both p65RelA and p50NF-kB1 activation and binding to DNA. These effects are specific as neither extracellular glucose levels nor fenoldopam affects the activation of the non-canonical NF-kB proteins RelB, p52NF-kB2 and c-Rel. Thus, fenoldopam can inhibit TNF production in splenocytes at high glucose concentrations, and can provide therapeutic advantages for treating diabetic patients with sepsis.

*In vivo*, treatment with fenoldopam attenuates serum TNF levels in diabetic mice with sepsis by inhibiting splenic TNF production. Given that NF-kB proteins are regulated by phosphorylation, we also analyzed p65NF-kB phosphorylation in the organs of the septic mice. Again, the most significant effects were found in the spleen where endotoxin increases p65NF-kB phosphorylation at serine 536 by over fourfold, and fenoldopam inhibits this phosphorylation by over threefold in the spleen without affecting the lung or liver. This specific inhibition of the canonical NF-kB pathway in the spleen can have clinical implications because NF-kB modulates cytokine production the spleen, and it protects parenchyma cells from cytotoxicity in other organs (66–68). The most characteristic example is that p65RelA (69–71) and IKKβ (72–74) knockout mice exhibit massive fetal hepatocyte apoptosis and embryonic death. These studies indicate that p65RelA can prevent hepatocyte apoptosis (73, 74), and thus ubiquitous NF-kB inhibition may not generate an overall beneficial effect especially in the liver, unless the therapy targets specific organs or immune cells (75). Therefore, fenoldopam may provide therapeutic advantages for diabetic patients with sepsis due to its potential to specifically inhibit NF-kB in the spleen. Furthermore, fenoldopam also attenuates hyperglycemia. These results have significant clinical implications because although hyperglycemia is especially relevant in diabetic patients, sepsis and other critical conditions such as hemorrhage, ischemia and trauma induce insulin resistant hyperglycemia in both diabetic and non-diabetic patients (76, 77). Sepsis is a complex process, and successful therapeutic treatments for sepsis may require controlling both immune and metabolic alterations. Given that hyperglycemia worsens systemic inflammation, organ function and mortality in sepsis (17–29), dopaminergic agonists such as fenoldopam may provide therapeutic advantages for both metabolic and immune alterations in sepsis and other critical conditions that induces insulin resistant hyperglycemia. Treatment with fenoldopam, started 15 h after the CLP, improves survival of diabetic mice with established polymicrobial peritonitis. By comparison with other strategies, administration of anti-TNF antibodies increased the mortality when administered after cecal perforation (78). Anti-macrophage MIF antibodies (79) or lysophosphatidylcholine (80) are ineffective if administered more than 8 or 10 h after the induction of peritonitis (79, 81). Our studies indicate that diabetes and glycemia affect the pathogenesis of sepsis and the efficacy of anti-inflammatory strategies. These results warrant further studies in other experimental models of diabetes and in other experimental groups including aging population Together, these results suggest that dopaminergic agonist type 1 can control systemic inflammation and provide therapeutic advantages for treating diabetic patients with sepsis in a clinically relevant time frame.

## Data Availability Statements

The raw data supporting the conclusions of this manuscript will be made available by the authors, without undue reservation, to any qualified researcher.

## Ethics Statement

All experimental procedures adhered to The Guide for the Care and Use of Laboratory Animals by the National Academy of Sciences and published by the National Institutes of Health (Copyright © 1996 by the National Academy of Sciences), and were approved by the Institutional Animal Care & Use Committee of the Rutgers New Jersey Medical School.

## Author Contributions

EF and RS performed all cellular experiments. ZL performed animal experiments. BJ performed animal experiments, figures preparation, and article editing. ZS contributed to experiment interpretation and article editing. LU directed the study and wrote the article. All authors approved the article before submission.

## Conflict of Interest Statement

The authors declare that the research was conducted in the absence of any commercial or financial relationships that could be construed as a potential conflict of interest.
